# Supplementary material for: The GPR120 agonist TUG‐891 promotes metabolic health by stimulating mitochondrial respiration in brown fat
Source: EMBO Mol Med. 2018 Jan 17;10(3):e8047. doi: 10.15252/emmm.201708047 (PMC5840546; doi:10.15252/emmm.201708047)
Supplement: Supplementary file 1 — Appendix [file EMMM-10-e8047-s001.pdf]

The GPR120 agonist TUG-891 promotes metabolic health by stimulating mitochondrial respiration in brown fat

Appendix

Table of contents:

Appendix Table S1

Appendix Table S2

Appendix Table S3

Appendix Table S4

Appendix Table S5

Appendix Figure S1 and figure legend

Appendix Figure S2 and figure legend

Appendix Figure S3 and figure legend

Appendix Figure S4 and figure legend

Appendix Figure S5 and figure legend

Appendix Figure S6 and figure legend

Appendix Figure S7 and figure legend

Appendix Figure S8 and figure legend

Appendix Figure S9 and figure legend

Appendix Table S1. Primer sequences for qRT-PCR.

Gene	Primer sequence		Product length (bp)
Aacs	Forward<br>Reverse	5'- CTGTCAGTGCTGGAGGAGAA -3'<br>5'- TGGCCCATGAAACAGGAGAT -3'	191
Acaa2	Forward<br>Reverse	5'- AGAAGGCCCTGGATCTTGAC -3'<br>5'- CTCCAATGCAAGCTGATCCC -3'	162
Acc1	Forward<br>Reverse	5'- TGTCCACCCAAGCATTTCTTC -3'<br>5'- CATCCAACACCAGTTCAGTATACGT -3'	75
Acc2	Forward<br>Reverse	5'- ACTTTGACCTGACCGCTGTG -3'<br>5'- CTGAGTGCCGGATAATGGC -3'	129
Acsl1	Forward<br>Reverse	5'- CAGAACCCGAAGATCTTGCG -3'<br>5'- CGGTCTCAAACATATGGGCG -3'	192
Adcy4	Forward<br>Reverse	5'- CCTCATTGCCCGCCTTTATC -3'<br>5'- GTCTCAGTCTCCTCTCGCTC -3'	195
Adhfe1	Forward<br>Reverse	5'- CCAGCTCCCTCCTGTACAAA -3'<br>5'- CCCACAGCAACATAGGCATC -3'	159
Adrb3	Forward<br>Reverse	5'- ATCGTGTCCGCTGCCGT -3'<br>5'- ATCTGCCCCTACACGCCAC -3'	63
Aldh2	Forward<br>Reverse	5'- GAGCAGAGCCATGTCATGTG -3'<br>5'- TGTCACACATCCAGGCATCT -3'	218
aP2	Forward<br>Reverse	5'- ACACCGAGATTTCCTTCAAACTG -3'<br>5'- CCATCTAGGGTTATGATGCTCTTCA -3'	88
Apoc1	Forward<br>Reverse	5'- GAGGGCGGTGGTGAATACTA -3'<br>5'- ATGCTCTCCAATGTTCCGGA -3'	183
Atgl	Forward<br>Reverse	5'- GCCAATGTCTGCAGCACATT -3'<br>5'- CATAGCGCACCCCTTGGA -3'	73
Ccl2 (Mcp1)	Forward<br>Reverse	5'- CAGGTCCCTGTCATGCTTCT -3'<br>5'- GAGTGGGGCGTTAACTGCAT -3'	93
Ccl5 (Rantes)	Forward<br>Reverse	5'- GCAAGTGCTCCAATCTTGCA -3'<br>5'- CTTCTCTGGGTTGGCACACA -3'	71
Ccna	Forward<br>Reverse	5'- CTGAAGGCCGGGAACGTG -3'<br>5'- CCTTAAGAGGAGCAACCCGT -3'	73
Ccnb	Forward<br>Reverse	5'- AAATTGCAGCTGGGGCTTTC -3'<br>5'- TGCAGAGTTGGTGTCCATTCA -3'	70
Cd36	Forward<br>Reverse	5'- GATGTGGAACCCATAACTGGA -3'<br>5'- GGCTTGACCAATATGTTGACC -3'	71
Cd68	Forward<br>Reverse	5'- CCAATTCAGGGTGGAAGAAA -3'<br>5'- GAGAGAGACAGGTGGGGATG -3'	104
Cidea	Forward<br>Reverse	5'- CACGCATTTCATGATCTTGGA -3'<br>5'- GTTGCTTGCAGACTGGGACAT -3'	74
Ctgf	Forward<br>Reverse	5'- AGCTGGGAGAACTGTGTACG -3'<br>5'- GCCAAATGTGTCTTCCAGTC -3'	380
Dgat2	Forward<br>Reverse	5'- TCGCGAGTACCTGATGTCTG -3'<br>5'- CTTCAGGGTGACTGCGTTCT -3'	160
Fasn	Forward<br>Reverse	5'- TGCGGAAACTTCAGGAAATGT -3'<br>5'- AGAGACGTGTCACTCCTGGACTT -3'	82
Gadd45a	Forward<br>Reverse	5'- GCTGCCAAGCTGCTCAAC -3'<br>5'- TCGTCGTCTTCGTCAGCA -3'	71
G0s2	Forward<br>Reverse	5'- AGTGCTGCCTCTCTTCCCAC -3'<br>5'- TTTCCATCTGAGCTCTGGGC -3'	65
Glut1	Forward<br>Reverse	5'- GACGGGCCGCCTCATGTTGG -3'<br>5'- GCTCTCCGTAGCGGTGGTTCC -3'	140

Glut4	Forward<br>Reverse	5’- CTATTCAACCAGCATCTTCGAG -3’<br>5’- CTACTAAGAGCACCGAGACC -3’	110
Gpr120	Forward<br>Reverse	5’- CCCCTCTGCATCTTGTTCC -3’<br>5’- GATTTCTCCTATGCGGTTGG -3’	102
Gys2	Forward<br>Reverse	5’- ATCCTTTCTCGTGCCAGGAA -3’<br>5’- GCGGTGGTATATCTGCCTCT -3’	159
Hsl	Forward<br>Reverse	5’- CGAGACAGGCCTCAGTGTGA -3’<br>5’- TCTGGGTCTATGGCGAATCG -3’	66
Il6	Forward<br>Reverse	5’- CTCTGGGAAATCGTGGAAAT -3’<br>5’- CCAGTTTGGTAGCATCCATC -3’	134
Insr	Forward<br>Reverse	5’- CTACAGTGTTCGAGTCCGGG -3’<br>5’- TGGCAATATTTGATGGGACATCT -3’	107
L19	Forward<br>Reverse	5’- GGAAAAAGAAGGTCTGGTTGGA -3’<br>5’- TGATCTGCTGACGGGAGTTG -3’	72
Lgals3	Forward<br>Reverse	5’- CAACCATCGGATGAAGAACC -3’<br>5’- TTCCCACTCCTAAGGCACAC -3’	141
Lpl	Forward<br>Reverse	5’- CAAGACCTTCGTGGTGATCCA -3’<br>5’- GTACAGGGCGGCCACAAGT -3’	82
Mki67	Forward<br>Reverse	5’- ACAGGCTCCGTACTTTCCAA -3’<br>5’- ACTGGATAGCACTTTTTCTCCCAA -3’	120
Mlxipl	Forward<br>Reverse	5’- CCCTCAGACACCCACATCTT -3’<br>5’- TCAGAAAGGGGTTGGGATCC -3’	209
Murf1	Forward<br>Reverse	5’- TGTGCAAGGAACACGAAGAC -3’<br>5’- CCAGCATGGAGATGCAGTTA -3’	171
Myog	Forward<br>Reverse	5’- CCCAACCCAGGAGATCATTT -3’<br>5’- GTCTGGGAAGGCAACAGACA -3’	117
Paqr9	Forward<br>Reverse	5’- GGTTTGCGTGGAGTTTCTGT -3’<br>5’- TGTTTCACCTCCCATCTCCC -3’	172
Pgc1α	Forward<br>Reverse	5’- GATGGCACGCAGCCCTAT -3’<br>5’- CTCGACACGGAGAGTTAAAGGAA -3’	70
Pnpla3	Forward<br>Reverse	5’- ACCTGAGAGCCTGCAATCTT -3’<br>5’- AACAGAACCCTTCCCAGAGG -3’	217
Pgc1α	Forward<br>Reverse	5’- GATGGCACGCAGCCCTAT -3’<br>5’- CTCGACACGGAGAGTTAAAGGAA -3’	70
Pparα	Forward<br>Reverse	5’- ATGCCAGTACTGCCGTTTTC -3’<br>5’- GGCCTTGACCTTGTTCATGT -3’	220
Pparγ	Forward<br>Reverse	5’- TTGTAGAGTGCCAGGTGCTG -3’<br>5’- CCTCCATAGCTCAGGTGGAA -3’	151
Scd1	Forward<br>Reverse	5’- CCCCTGCGGATCTTCCTTAT -3’<br>5’- AGGGTCGGCGTGTGTTTCT -3’	114
Scd2	Forward<br>Reverse	5’- AGCGGGCTGCAGAAACTTAG -3’<br>5’- GGCTGAGTAAGCGCCAGAGAT -3’	148
Sncg	Forward<br>Reverse	5’- CAAGGAAGGTGTTGTGGGTG -3’<br>5’- CTTGTTGGCCACTGTGTTGA -3’	208
Srebp1c	Forward<br>Reverse	5’- ATGCCATGGGCAAGTACACA -3’<br>5’- ATAGCATCTCCTGCGCACTC -3’	91
Tnfα	Forward<br>Reverse	5’- ATGAGAAGTTCCCAAATGGC -3’<br>5’- CTCCACTTGGTGGTTTGCTA -3’	125
Ucp1	Forward<br>Reverse	5’- TACCCAAGCGTACCAAGCTG -3’<br>5’- ACCCGAGTCGCAGAAAAGAA -3’	97
Vegf	Forward<br>Reverse	5’- CATCTTCAAGCCGTCCTGTGT -3’<br>5’- CTCCAGGGCTTCATCGTTACA -3’	67
Vldlr	Forward<br>Reverse	5’- GCCCTGAACAGTGCCATATG -3’<br>5’- CATCACTGCCATCGTCACAG -3’	243

β-Actin	Forward<br>Reverse	5'- GCAGGAGTACGATGAGTCCG -3'<br>5'- ACGCAGCTCAGTAACAGT -3'	74
β-2 microglobulin	Forward<br>Reverse	5'- TGACCGGCTTGTATGCTATC -3'<br>5'- CAGTGTGAGCCAGGATATAG -3'	222

Appendix Table S2. Culture medium compounds and their concentrations.

Compound	ImBA Induction	ImBA Maintenance	ImWA Induction	ImWA Maintenance
Thyroid hormone T_3	1 nM	1 nM	0.1 nM	0.1 nM
Insulin	1 μg/ml	1 μg/ml	5 μg/ml	5 μg/ml
Biotin	16 μM	16 μM	16 μM	16 μM
Pantothenate	1.8 μM	1.8 μM	1.8 μM	1.8 μM
Ascorbic acid	100 μM	100 μM	100 μM	100 μM
IBMX	0.5 mM	-	0.5 mM	-
Dexamethasone	250 nM	-	250 nM	-
Indomethacin	125 μM	-	-	-
Cortisol	-	-	100 nM	-
Rosiglitazone	-	-	1 μM	-

ImBA: immortalized brown adipocytes, ImWA: immortalized white adipocytes.

Appendix Table S3. Genes upregulated or downregulated in the absence of GPR120.

Table S3A. Genes upregulated in BAT from GPR120 KO versus WT mice.

Gene Symbol	Gene Name	FC	Adj P Value
Sncg	synuclein, gamma	1.99	0.037
Xlr4a	X-linked lymphocyte-regulated 4A	1.86	0.047
Npm3-ps1	nucleoplasmin 3, pseudogene 1	1.75	0.039
Cd52	CD52 antigen	1.71	0.014
Rarres2	retinoic acid receptor responder (tazarotene induced) 2	1.71	0.021
Hist1h2ap	histone cluster 1, H2ap	1.68	0.011
Wdfy1	WD repeat and FYVE domain containing 1	1.67	0.021
Cxcl9	chemokine (C-X-C motif) ligand 9	1.65	0.011
Hist1h2ad	histone cluster 1, H2ad	1.62	0.011
Ccl11	chemokine (C-C motif) ligand 11	1.62	0.023
Npm3	nucleoplasmin 3	1.52	0.040
Hebp1	heme binding protein 1	1.52	0.013
Coro1a	coronin, actin binding protein 1A	1.52	0.008
Hn1	hematological and neurological expressed sequence 1	1.50	0.023
Rps12-ps24	Ribosomal protein S12, pseudogene 24	1.49	0.028
Cd74	CD74 antigen (invariant polypeptide of major histocompatibility complex, class II antigen-associated)	1.48	0.045
Hist1h2ai	histone cluster 1, H2ai	1.48	0.022
Sae1	SUMO1 activating enzyme subunit 1	1.48	0.009

Gm11425	predicted gene 11425	1.46	0.034
Psmb9	proteasome (prosome, macropain) subunit, beta type 9 (large multifunctional peptidase 2)	1.45	0.005
Slamf9	SLAM family member 9	1.45	0.031
Pigp	phosphatidylinositol glycan anchor biosynthesis, class P	1.45	0.034
Maged2	melanoma antigen, family D, 2	1.45	0.011
H2-Eb1	histocompatibility 2, class II antigen E beta	1.44	0.034
Hist1h2af	histone cluster 1, H2af	1.44	0.027
Dus4l	dihydrouridine synthase 4-like (S. cerevisiae)	1.43	0.029
Wbp5	WW domain binding protein 5	1.42	0.037
Rpl12	ribosomal protein L12	1.41	0.031
Rps5	ribosomal protein S5	1.41	0.046
1110059E24Rik	RIKEN cDNA 1110059E24 gene	1.41	0.003
Mettl20	Methyltransferase like 20	1.41	0.025
Anxa2	annexin A2	1.40	0.004
Mgst3	microsomal glutathione S-transferase 3	1.39	0.026
Nsmce1	non-SMC element 1 homolog (S. cerevisiae)	1.39	0.021
Hist1h2an	histone cluster 1, H2an	1.39	0.027
Lsm5	LSM5 homolog, U6 small nuclear RNA associated (S. cerevisiae)	1.38	0.034
Mpeg1	macrophage expressed gene 1	1.38	0.025
Ubxn6	UBX domain protein 6	1.38	0.023
Rps12	ribosomal protein S12	1.38	0.026
Evi2a	ecotropic viral integration site 2a	1.38	0.034
Arhgdib	Rho, GDP dissociation inhibitor (GDI) beta	1.38	0.036
Fbn1	fibrillin 1	1.37	0.049
Rps26	ribosomal protein S26	1.37	0.034
Rhoj	ras homolog gene family, member J	1.37	0.015
Ugt1a10	UDP glycosyltransferase 1 family, polypeptide A10	1.37	0.034
Mgst3	microsomal glutathione S-transferase 3	1.36	0.009
Tyrobp	TYRO protein tyrosine kinase binding protein	1.36	0.031
Ebpl	emopamil binding protein-like	1.36	0.016
Arpc3	actin related protein 2/3 complex, subunit 3	1.36	0.033
Siva1	SIVA1, apoptosis-inducing factor	1.35	0.040

Table S3B. Genes downregulated in BAT from GPR120 KO versus WT mice.

**Gene Symbol**	**Gene Name**	**FC**	**Adj P Value**
O3far1	omega-3 fatty acid receptor 1	0.35	0.002
Pnpla3	patatin-like phospholipase domain containing 3	0.38	0.016
Hspa8	heat shock protein 8	0.41	0.001
Cyp2b10	cytochrome P450, family 2, subfamily b, polypeptide 10	0.43	0.038
Adrbk2	adrenergic receptor kinase, beta 2	0.55	0.004
Vegfa	vascular endothelial growth factor A	0.55	0.003
Apoc1	apolipoprotein C-I	0.55	0.004
Mlxipl	MLX interacting protein-like	0.56	0.007
Hspd1	heat shock protein 1 (chaperonin)	0.56	0.017
Luc7l3	LUC7-like 3 (S. cerevisiae)	0.58	0.015
Per2	period homolog 2 (Drosophila)	0.60	0.011
Atl2	atlastin GTPase 2	0.60	0.034
Neat1	nuclear paraspeckle assembly transcript 1 (non-protein	0.61	0.050

	coding)		
Acsl1	acyl-CoA synthetase long-chain family member 1	0.61	0.041
Ggnbp1	gametogenetin binding protein 1	0.61	0.011
Slc4a4	solute carrier family 4 (anion exchanger), member 4	0.61	0.014
Per2	period homolog 2 (Drosophila)	0.61	0.021
Rnf44	ring finger protein 44	0.62	0.023
Flcn	folliculin	0.62	0.028
Slc25a20	solute carrier family 25 (mitochondrial carnitine/acylcarnitine translocase), member 20	0.62	0.025
Atl2	atlastin GTPase 2	0.62	0.047
Hnrpdl	heterogeneous nuclear ribonucleoprotein D-like	0.62	0.011
Srsf2	serine/arginine-rich splicing factor 2	0.63	0.016
Slc38a2	solute carrier family 38, member 2	0.63	0.016
Gys2	glycogen synthase 2	0.63	0.023
Ddx17	DEAD (Asp-Glu-Ala-Asp) box polypeptide 17	0.63	0.014
Gm3308	predicted gene 3308	0.64	0.021
Gm10621	predicted gene 10621	0.64	0.004
Ccrn4l	CCR4 carbon catabolite repression 4-like (S. cerevisiae)	0.64	0.024
Actb	actin, beta	0.64	0.021
Aspg	asparaginase homolog (S. cerevisiae)	0.64	0.021
Clasrp	CLK4-associating serine/arginine rich protein	0.64	0.013
Pparγc1b	peroxisome proliferative activated receptor, gamma, coactivator 1 beta	0.64	0.029
Apoc1	apolipoprotein C-I	0.65	0.034
Hnrnpa2b1	heterogeneous nuclear ribonucleoprotein A2/B1	0.65	0.017
Adhfe1	alcohol dehydrogenase, iron containing, 1	0.65	0.028
Klf9	Kruppel-like factor 9	0.65	0.029
Aacs	acetoacetyl-CoA synthetase	0.66	0.039
Vegfα	vascular endothelial growth factor A	0.66	0.003
Srrm2	serine/arginine repetitive matrix 2	0.66	0.013
2810403A07Rik	RIKEN cDNA 2810403A07 gene	0.67	0.011
Hsp90ab1	heat shock protein 90 alpha (cytosolic), class B member 1	0.67	0.046
Gtf3c2	general transcription factor IIIC, polypeptide 2, beta	0.67	0.011
Lgals4	lectin, galactose binding, soluble 4	0.67	0.011
Paqr9	progestin and adipoQ receptor family member IX	0.67	0.030
Arap3	ArfGAP with RhoGAP domain, ankyrin repeat and PH domain 3	0.68	0.030
Slc1a5	solute carrier family 1 (neutral amino acid transporter), member 5	0.68	0.001
Fam126b	family with sequence similarity 126, member B	0.68	0.022
Slc1a5	solute carrier family 1 (neutral amino acid transporter), member 5	0.68	0.003
Gm2589	predicted gene 2589	0.68	0.012

FC: fold change.

Appendix Table S4. Functional annotation clustering of genes upregulated or downregulated in the absence of GPR120.

Table S4A. DAVID (P=0.05), upregulated in GPR120 KO, 7 clusters with terms with FDR < 0.01.

**Annotation Cluster 1**	**Enrichment Score: 23.571326366746383**	
**Category**	**Term**	**FDR**
GOTERM_MF_FAT	GO:0003735~structural constituent of ribosome	3.18E-27
SP_PIR_KEYWORDS	ribonucleoprotein	4.17E-26
KEGG_PATHWAY	mmu03010:Ribosome	6.46E-25
GOTERM_CC_FAT	GO:0005840~ribosome	3.05E-24
SP_PIR_KEYWORDS	ribosomal protein	3.34E-24
GOTERM_CC_FAT	GO:0030529~ribonucleoprotein complex	2.50E-23
GOTERM_BP_FAT	GO:0006412~translation	2.83E-21
GOTERM_MF_FAT	GO:0005198~structural molecule activity	1.14E-10
GOTERM_CC_FAT	GO:0033279~ribosomal subunit	3.05E-08
**Annotation Cluster 2**	**Enrichment Score: 5.009748166504468**	
**Category**	**Term**	**FDR**
GOTERM_CC_FAT	GO:0043228~non-membrane-bounded organelle	5.14E-05
GOTERM_CC_FAT	GO:0043232~intracellular non-membrane-bounded organelle	5.14E-05
**Annotation Cluster 3**	**Enrichment Score: 3.6450159174973065**	
**Category**	**Term**	**FDR**
GOTERM_BP_FAT	GO:0006396~RNA processing	3.92 E-03
**Annotation Cluster 6**	**Enrichment Score: 3.083826976916491**	
**Category**	**Term**	**FDR**
GOTERM_BP_FAT	GO:0048002~antigen processing and presentation of peptide antigen	1.72E-08
GOTERM_BP_FAT	GO:0002478~antigen processing and presentation of exogenous peptide antigen	7.16E-06
GOTERM_BP_FAT	GO:0019882~antigen processing and presentation	9.29E-06
GOTERM_BP_FAT	GO:0019884~antigen processing and presentation of exogenous antigen	1.00E-05
GOTERM_BP_FAT	GO:0002495~antigen processing and presentation of peptide antigen via MHC class II	1.38E-05
GOTERM_BP_FAT	GO:0019886~antigen processing and presentation of exogenous peptide antigen via MHC class II	1.38E-05
GOTERM_BP_FAT	GO:0002504~antigen processing and presentation of peptide or polysaccharide antigen via MHC class II	1.33E-04
**Annotation Cluster 15**	**Enrichment Score: 2.4342394169368364**	
**Category**	**Term**	**FDR**
GOTERM_BP_FAT	GO:0002443~leukocyte mediated immunity	1.62E-03
GOTERM_BP_FAT	GO:0002252~immune effector process	3.34E-03
GOTERM_BP_FAT	GO:0050778~positive regulation of immune response	4.05E-03
GOTERM_BP_FAT	GO:0048584~positive regulation of response to stimulus	6.99E-03
**Annotation Cluster 17**	**Enrichment Score: 2.306287251432978**	

**Category**	**Term**	**FDR**
GOTERM_BP_FAT	GO:0050778~positive regulation of immune response	4.05E-03
**Annotation Cluster 30**	**Enrichment Score: 1.6629313948276978**	
**Category**	**Term**	**FDR**
KEGG_PATHWAY	mmu05322:Systemic lupus erythematosus	6.07E-03

Table S4B. DAVID (P=0.05), downregulated in GPR120 KO, 11 clusters with terms with FDR < 0.01.

**Annotation Cluster 1**	**Enrichment Score: 18.04039228677734**	
**Category**	**Term**	**FDR**
GOTERM_CC_FAT	GO:0005739~mitochondrion	1.54E-21
SP_PIR_KEYWORDS	mitochondrion	3.77E-18
SP_PIR_KEYWORDS	transit peptide	1.19E-15
UP_SEQ_FEATURE	transit peptide: Mitochondrion	4.19E-12
GOTERM_CC_FAT	GO:0044429~mitochondrial part	1.58E-10
**Annotation Cluster 2**	**Enrichment Score: 10.830871851860504**	
**Category**	**Term**	**FDR**
SP_PIR_KEYWORDS	mitochondrion	3.77E-18
GOTERM_CC_FAT	GO:0031090~organelle membrane	1.56E-11
GOTERM_CC_FAT	GO:0044429~mitochondrial part	1.58E-10
GOTERM_CC_FAT	GO:0031966~mitochondrial membrane	9.36E-08
GOTERM_CC_FAT	GO:0019866~organelle inner membrane	1.02E-07
GOTERM_CC_FAT	GO:0031967~organelle envelope	6.85E-07
GOTERM_CC_FAT	GO:0031975~envelope	8.04E-07
GOTERM_CC_FAT	GO:0005740~mitochondrial envelope	1.09E-06
GOTERM_CC_FAT	GO:0005743~mitochondrial inner membrane	1.34E-06
**Annotation Cluster 3**	**Enrichment Score: 9.454393303217916**	
**Category**	**Term**	**FDR**
GOTERM_MF_FAT	GO:0000166~nucleotide binding	1.68E-17
GOTERM_MF_FAT	GO:0017076~purine nucleotide binding	1.51E-08
GOTERM_MF_FAT	GO:0001882~nucleoside binding	4.75E-08
GOTERM_MF_FAT	GO:0001883~purine nucleoside binding	5.33E-08
GOTERM_MF_FAT	GO:0030554~adenyl nucleotide binding	9.68E-08
GOTERM_MF_FAT	GO:0032553~ribonucleotide binding	5.60E-06
GOTERM_MF_FAT	GO:0032555~purine ribonucleotide binding	5.60E-06
SP_PIR_KEYWORDS	nucleotide-binding	6.45E-06
SP_PIR_KEYWORDS	ATP-binding	1.09E-05
GOTERM_MF_FAT	GO:0005524~ATP binding	1.81E-05
GOTERM_MF_FAT	GO:0032559~adenyl ribonucleotide binding	3.84E-05
**Annotation Cluster 4**	**Enrichment Score: 8.909158524499384**	
**Category**	**Term**	**FDR**
GOTERM_CC_FAT	GO:0031974~membrane-enclosed lumen	7.00E-11
GOTERM_CC_FAT	GO:0070013~intracellular organelle lumen	9.83E-11
GOTERM_CC_FAT	GO:0043233~organelle lumen	1.20E-10
GOTERM_CC_FAT	GO:0031981~nuclear lumen	2.30E-05
GOTERM_CC_FAT	GO:0016607~nuclear speck	6.01E-05

GOTERM_CC_FAT	GO:0016604~nuclear body	4.99E-04
GOTERM_CC_FAT	GO:0005654~nucleoplasm	5.15E-03
**Annotation Cluster 5**	**Enrichment Score: 8.144022074819771**	
**Category**	**Term**	**FDR**
GOTERM_CC_FAT	GO:0044429~mitochondrial part	1.58E-10
GOTERM_CC_FAT	GO:0005759~mitochondrial matrix	2.59E-03
GOTERM_CC_FAT	GO:0031980~mitochondrial lumen	2.59E-03
**Annotation Cluster 6**	**Enrichment Score: 8.114976494819215**	
**Category**	**Term**	**FDR**
INTERPRO	IPR012677:Nucleotide-binding, alpha-beta plait	1.94E-09
INTERPRO	IPR000504:RNA recognition motif, RNP-1	6.55E-09
SP_PIR_KEYWORDS	RNA-binding	2.95E-08
SMART	SM00360:RRM	9.91E-08
GOTERM_MF_FAT	GO:0003723~RNA binding	1.84E-06
UP_SEQ_FEATURE	domain:RRM 1	1.36E-03
UP_SEQ_FEATURE	domain:RRM 2	1.36E-03
**Annotation Cluster 7**	**Enrichment Score: 5.577820081203797**	
**Category**	**Term**	**FDR**
GOTERM_BP_FAT	GO:0016071~mRNA metabolic process	2.99E-05
GOTERM_BP_FAT	GO:0006397~mRNA processing	6.49E-05
SP_PIR_KEYWORDS	mRNA processing	6.53E-05
GOTERM_BP_FAT	GO:0008380~RNA splicing	8.83E-05
GOTERM_BP_FAT	GO:0006396~RNA processing	3.05E-04
SP_PIR_KEYWORDS	mRNA splicing	4.12E-04
**Annotation Cluster 8**	**Enrichment Score: 4.573455401588822**	
**Category**	**Term**	**FDR**
SP_PIR_KEYWORDS	fatty acid metabolism	1.78E-03
**Annotation Cluster 9**	**Enrichment Score: 4.428903499912428**	
**Category**	**Term**	**FDR**
SP_PIR_KEYWORDS	Flavoprotein	5.53E-05
GOTERM_MF_FAT	GO:0050662~coenzyme binding	2.56E-03
SP_PIR_KEYWORDS	FAD	2.78E-03
**Annotation Cluster 11**	**Enrichment Score: 3.7314344007433613**	
**Category**	**Term**	**FDR**
GOTERM_CC_FAT	GO:0005777~peroxisome	9.14E-04
GOTERM_CC_FAT	GO:0042579~microbody	9.14E-04
**Annotation Cluster 47**	**Enrichment Score: 1.4911957984288633**	
**Category**	**Term**	**FDR**
SP_PIR_KEYWORDS	nucleus	2.20E-04

FDR: false discovery rate.

Appendix Table S5. *P*-values from statistical tests.

Figure	***P*-value**		
	**Vehicle versus TUG-891**		
**1A**	1.5 wks: 0.0373 (*)<br>2.5 wks: 0.0047 (**)		
**1B**	0.75 wks: 0.0302 (*)<br>1.5 wks: 0.0016 (**)<br>2.5 wks: 0.0001(***)		
**1C**	2.5 wks: 0.0122 (*)		
**1D**	2 wks: 0.0020 (**)		
**1E**	Dark: 0.0045 (**)		
**1F**	Light: 0.0433 (*)<br>Dark: 0.0221 (*)		
**1G**	Dark: 0.0040 (*)		
	**Vehicle versus TUG-891**		
**2A**	0.0075 (**)		
**2B**	0.0014 (**)		
**2C**	0.0000 (***)		
**2D**	iBAT: 0.0002 (***)<br>gWAT: 0.0000 (***)<br>Liver: 0.0093 (*)		
	**Vehicle versus WT TUG-891**	**Vehicle versus KO TUG-891**	**WT TUG-891 versus KO TUG-891**
**3A**	12 d: 0.0163 (*)	n.s.	n.s.
**3B**	12 d: 0.0224 (*)	n.s.	n.s.
**3C**	6-12 d: 0.0094 (**)	6-12 d: 0.0260 (*)	n.s.
**3F**	Dark: 0.0349 (*)	n.s.	n.s.
	**Vehicle versus WT TUG-891**	**Vehicle versus KO TUG-891**	**WT TUG-891 versus KO TUG-891**
**4A**	iBAT: 0.0000 (***)<br>sBAT: 0.0000 (***)	iBAT: 0.0192 (*)<br>sBAT: 0.0001 (***)	iBAT: 0.0000 (***)<br>sBAT: 0.0000 (***)
**4B**	iBAT: 0.0290 (*)<br>sBAT: 0.0000 (***)	n.s.	iBAT: 0.006283 (**)<br>sBAT: 0.0000 (***)
**4C**	iBAT: 0.0000 (***)<br>sWAT: 0.0149 (*)	Liver: 0.0024 (**)	n.d.
	**Vehicle versus TUG-891**		
**5C**	*Sncg*: 0.0118 (*)<br>*Mlxipl*: 0.0368 (*)		
**5D**	*Glut4:* 0.0453 (*)<br>*Insr*: 0.0007 (***)<br>*Adcy4*: 0.0092 (**)		
**5E**	*Acc1*: 0.0353 (*)<br>*Acc2*: 0.0213 (*)<br>*Fas*: 0.0158 (*)<br>*Scd2*: 0.0340 (*)<br>*Hsl*: 0.0021 (**)<br>*Atgl*: 0.0353 (*)<br>*Pnpla3*: 0.0047 (**)<br>*Apoc1*: 0.0062 (**)		
	**Vehicle versus CL**		
**6A – *Gpr120***	0.0028 (**)		
**6A – *Ucp1***	0.0016 (**)		

	**Undiff versus Diff**	**Undiff versus Diff + CL**	**Diff versus Diff + CL**
**6B – *Gpr120***	n.d.	n.d.	BA: 0.0000 (***)
**6B – *Ucp1***	n.d.	n.d.	BA: 0.0000 (***)
	**WT vehicle versus WT TUG-891**	**WT vehicle versus KO vehicle**	**WT vehicle versus KO TUG-891**
**6C**	Day 0: n.d.<br>Day 7: 0.0015 (**)	Day 0: n.d.<br>Day 3: 0.0050 (**)<br>Day 7: 0.0001 (***)<br>Day 9: 0.0001 (***)	Day 0: n.d.<br>Day 3: 0.0052 (**)<br>Day 7: 0.0001 (***)<br>Day 9: 0.0001 (***)
**6D – *aP2***	Day 0: n.d.	Day 0: n.d.<br>Day 3: 0.099 (**)<br>Day 7: 0.0001 (***)<br>Day 9: 0.0001 (***)	Day 0: n.d.<br>Day 3: 0.0017 (**)<br>Day 7: 0.0001 (***)<br>Day 9: 0.0001 (***)
**6D – *Ucp1***	Day 0: n.d.	Day 0: n.d.<br>Day 7: 0.0001 (***)<br>Day 9: 0.0001 (***)	Day 0: n.d.<br>Day 7: 0.0001 (***)<br>Day 9: 0.0001 (***)
	**TUG-891 versus vehicle**	**TUG-891 versus TUG-891 + AH7614**	**TUG-891 + AH7614 versus vehicle**
**7A**	23 min: 0.0001 (***)<br>30 min: 0.0000 (***)<br>38 min: 0.0000 (***)<br>45 min: 0.0000 (***)	23 min: 0.0033 (##)<br>30 min: 0.0006 (###)<br>38 min: 0.0013 (##)<br>45 min: 0.0017 (##)	n.d.
	**TUG-891 versus vehicle**	**TUG-891 versus TUG-891 + BAPTA**	**TUG-891 + BAPTA versus vehicle**
**7D**	23 min: 0.0005 (***)<br>30 min: 0.0009 (***)<br>38 min: 0.0004 (***)<br>45 min: 0.0003 (***)<br>53 min: 0.0002 (***)<br>60 min: 0.0000 (***)	23 min: 0.0138 (#)<br>30 min: 0.0415 (#)<br>38 min: 0.0334 (#)<br>45 min: 0.0211 (#)<br>53 min: 0.0238 (#)<br>60 min: 0.0288 (#)	n.d.
	**Basal versus 10 min**		
**7F**	TUG-891: 0.0094 (**)		
	**Vehicle versus TUG-891**		
**S3A**	0.0483 (*)		
**S3F**	*Scd1*: 0.0001 (***)		
**S3G**	*Acc1:* 0.0455 (*)<br>*Scd1:* 0.0267 (*)<br>*Ccnb:* 0.0149 (*)		
**SGH**	*Dgat2:* 0.0441 (*)<br>*Ccna:* 0.0003 (***)<br>*Ccnb:* 0.0037 (**)<br>*Mki67:* 0.0010 (***)<br>*Ucp1:* 0.0157 (*)		
	**Vehicle versus TUG-891**		
**S4C**	0.0227 (*)		
	**WT vehicle versus WT TUG-891**	**WT vehicle versus KO vehicle**	**WT vehicle versus KO TUG-891**
**S6A**	Liver: 0.0000 (***)<br>iBAT: 0.0023 (**)	n.s.	Liver: 0.0000 (***)
**S6B**	n.s.	n.s.	Liver: 0.0444 (*)
	**Vehicle versus CL**		
**S8A**	0.0362 (*)		

**S8D**	0.0005 (***)		
	**WT TUG-891 versus UCP1 KO TUG-891**		
**S9A**	10 µM: 0.0092 (**)<br>20 µM: 0.0144 (*)<br>30 µM: 0.0337 (*)<br>40 µM: 0.0211 (*)<br>60 µM: 0.0143 (*)<br>90 µM: 0.0003 (***)<br>100 µM: 0.0002 (***)		
	**WT 1 mM GDP versus WT 3 mM GDP**		
**S9B**	20 µM: 0.0359 (*)		
	40 µM: 0.0402 (*)<br>50 µM: 0.0074 (**)<br>60 µM: 0.0364 (*)<br>80 µM: 0.0121 (*)<br>90 µM: 0.0064 (**)<br>100 µM: 0.0112 (*)<br>110 µM: 0.0214 (*)		
	**Vehicle versus TUG-891**	**Vehicle versus forskolin**	**TUG-891 versus forskolin**
**S9D**	n.s.	0.0211 (*)	n.d.

N.s.: non-significant, n.d: non-determined. All P-values are rounded to 4 decimals.

Appendix Figure S1. TUG-891 non-significantly affects gene expression in skeletal muscle. Expression of markers for inflammation (*Cd68* and *Lgals3*) fibrosis (*Ctgf*), atrophy (*Gadd45a* and *Murf1*) and regeneration (*Myog*) were determined in skeletal muscle tissue of mice treated with vehicle or TUG-891 by qRT-PCR. Data represent means ± SEM (*n* = 8 per group).

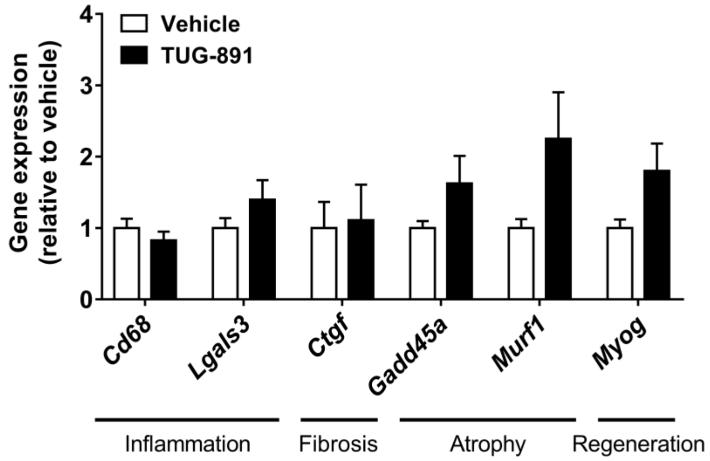

Appendix Figure S2. TUG-891 does not affect energy expenditure or physical activity levels. Energy expenditure (EE) (A) and physical activity (B) were determined by housing the mice individually in metabolic cages. Injection of TUG-891 or vehicle is indicated by dotted lines, and light and grey areas represent the light and dark phase, respectively. For bar graph analysis, mean results in the light and dark phase were calculated. Data represent means ± SEM (*n* = 8 per group).

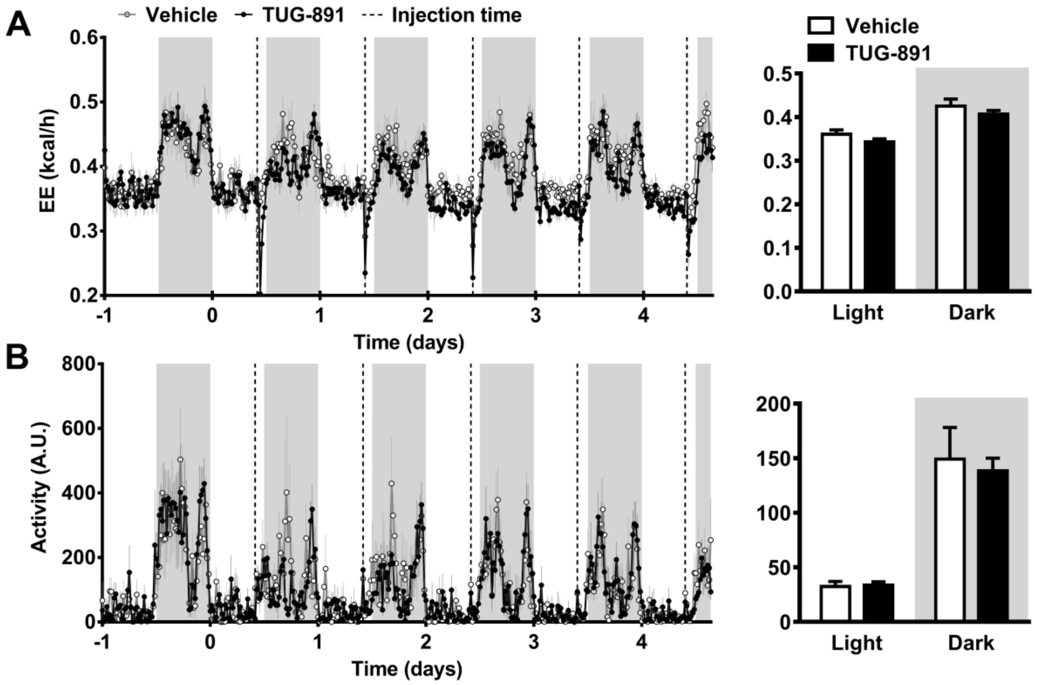

Appendix Figure S3. TUG-891 increases gene expression of markers for proliferation and thermogenesis in WAT. Plasma triglycerides (TG) (A), protein levels of UCP1, $pHSL_{563}$ and pPKA substrates in BAT (B-E), and gene expression markers for lipolysis, lipogenesis, proliferation and thermogenesis in BAT (F) and WAT (G-H) were determined in vehicle and TUG-891-treated mice. Data represent means ± SEM (*n* = 8 per group). * P < 0.05, ** P < 0.01, *** P < 0.001 compared to the vehicle group, according to the two-tailed unpaired Student's T test. The exact *P*-value for each significant difference can be found in Appendix Table S5.

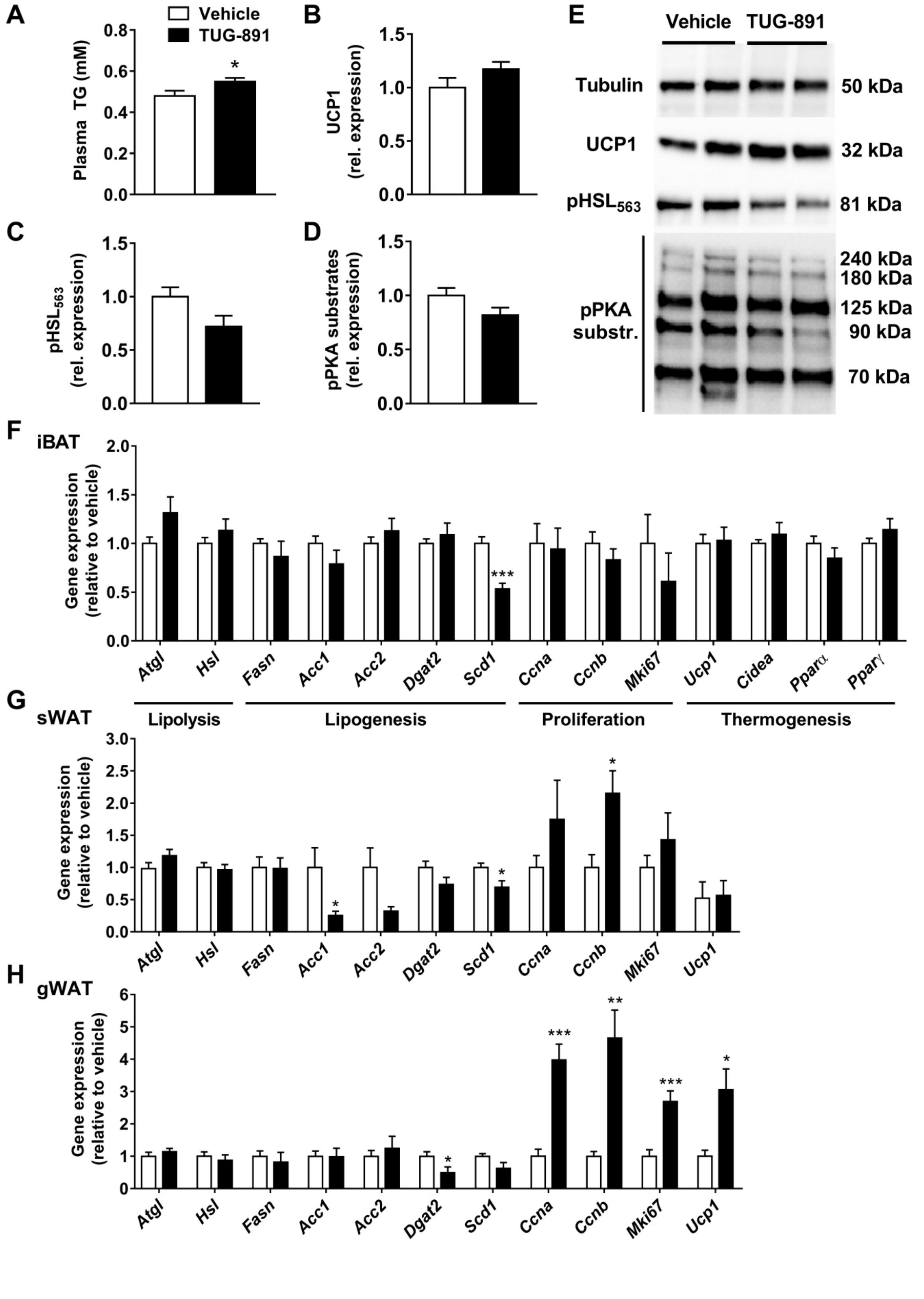

Appendix Figure S4. TUG-891 increases protein expression of UCP1 in gWAT. Representative images of UCP1 immunostained interscapular BAT (iBAT, A), subcutaneous WAT (sWAT, B) and gonadal WAT (gWAT, C) of mice treated with vehicle or the GPR120 agonist TUG-891. Stained slides were digitalized and relative UCP1 staining per area was analysed using ImageJ software. Data represent means ± SEM (n = 8 per group). * P < 0.05 compared to the vehicle group, according to the two-tailed unpaired Student's T test. The exact P-value for each significant difference can be found in Appendix Table S5.

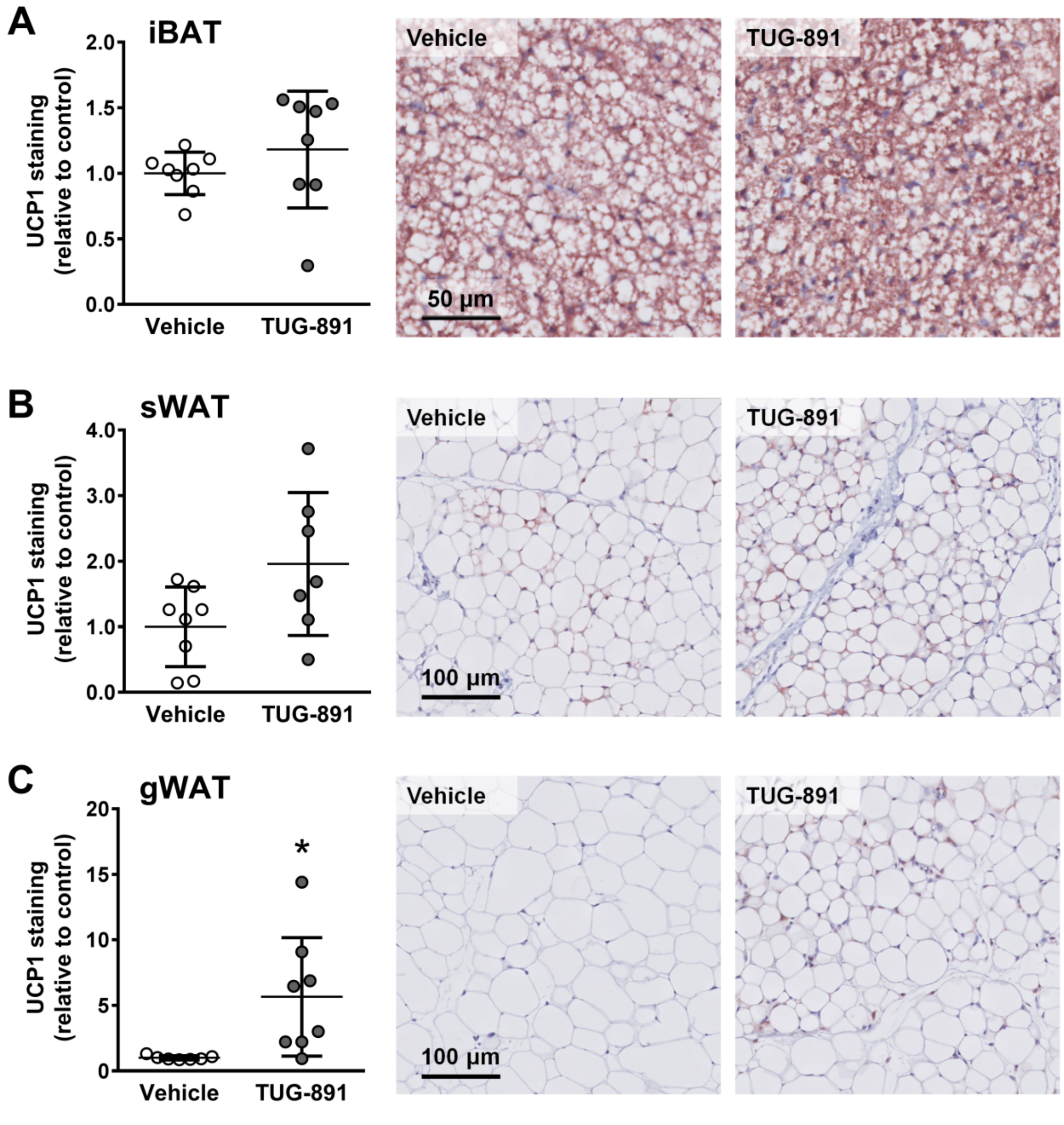

Appendix Figure S5. Respiratory exchange ratio and fat oxidation is similar between wild type and GPR120-deficient mice. Respiratory exchange ratio (RER) (A) and fat oxidation (B) was determined by housing GPR120 KO mice (*n* = 6) and WT littermates (*n* = 13) in metabolic cages. Light and grey areas represent the light and dark phase, respectively. For bar graph analysis, mean results in light and dark phase were calculated. Data represent means ± SEM.

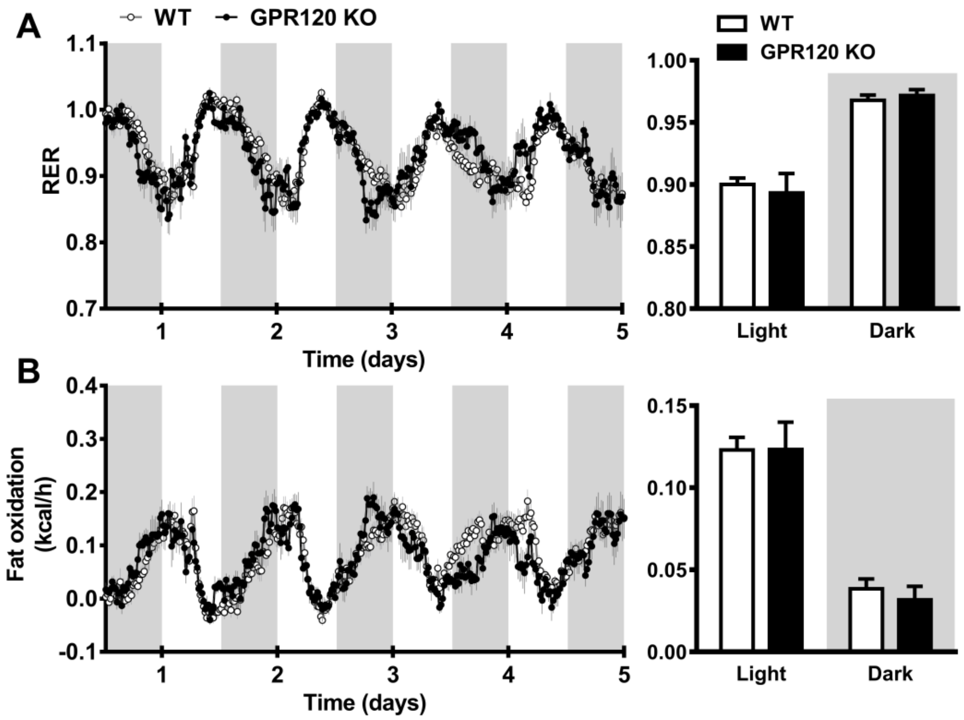

Appendix Figure S6. TUG-891 increases whole organ uptake of fatty acids by BAT. WT and GPR120 KO mice treated with vehicle or the GPR120 agonist TUG-891 were intravenously injected with glycerol tri[^{3}H]oleate ([^{3}H]TO)-labeled lipoprotein-like emulsion particles and [^{14}C]deoxyglucose ([^{14}C]DG). After 15 min, mice were sacrificed and uptake of [^{3}H]TO- and [^{14}C]DG-derived radioactivity (A-B) per whole organ was determined in organs that were dissected quantitatively, including gonadal WAT (gWAT) and interscapular BAT (iBAT). Data represent means ± SEM (n = 6-8 per group). * P < 0.05, ** P < 0.01, *** P < 0.001 compared to the vehicle group or indicated control group, according to two-way ANOVA with Tukey's post hoc test (A-B). The exact P-value for each significant difference can be found in Appendix Table S5.

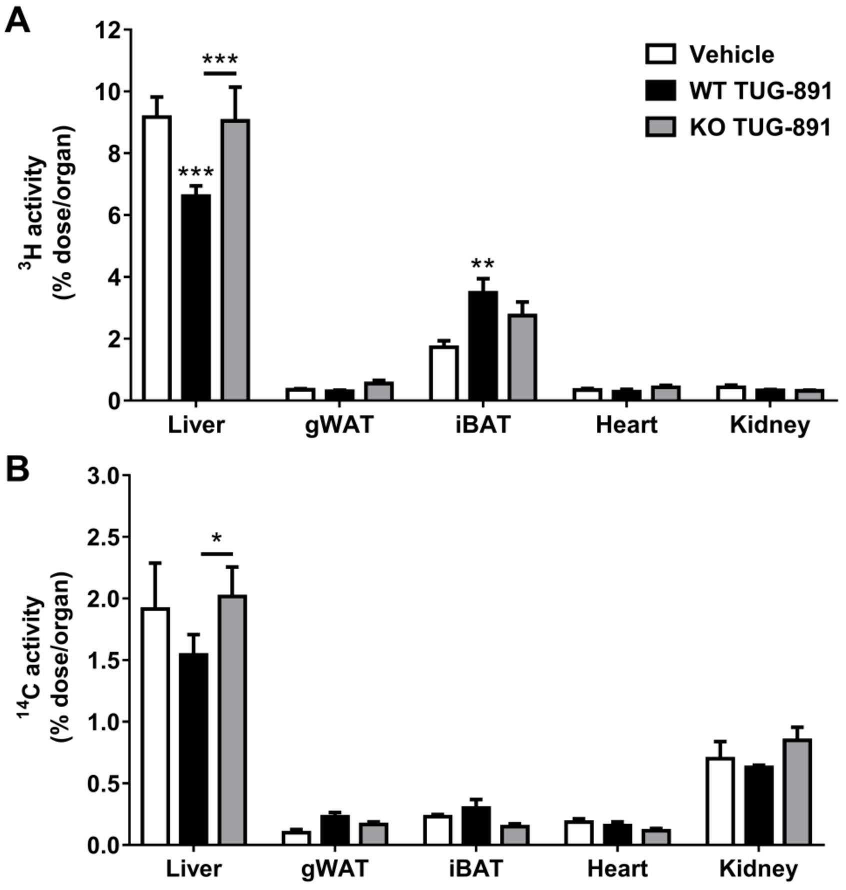

Appendix Figure S7. *Gpr120* is most highly expressed in BAT. *Gpr120* gene expression was determined in various tissues of FVB/N female mice, including brown adipose tissue (BAT), subcutaneous white adipose tissue (sWAT) and gonadal white adipose tissue (gWAT). Data represent means ± SEM ($n = 3$).

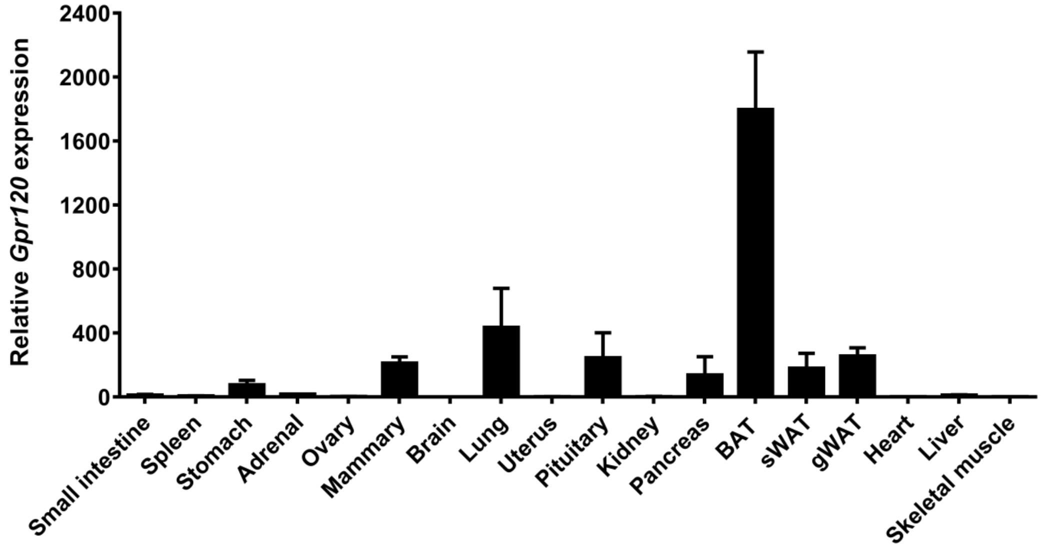

Appendix Figure S8. Gene expression profiles of *aP2*, *Cidea*, *Pref1* and *Adrb3* validate the immortalized murine brown adipocyte cell line. Immortalized murine brown adipocytes were differentiated for 0, 2, 4, 6, or 7 days after which expression of *aP2* (A), *Cidea* (B), *Pref1* (C), and *Adrb3* (D) was determined by qRT-PCR. On day 7, a subset of adipocytes was additionally stimulated with CL (10 µM). Data represent means ± SEM ($n = 3$). * $P < 0.05$, *** $P < 0.001$ compared to the vehicle group, according to the two-tailed unpaired Student's T test. The exact *P*-value for each significant difference can be found in Appendix Table S5.

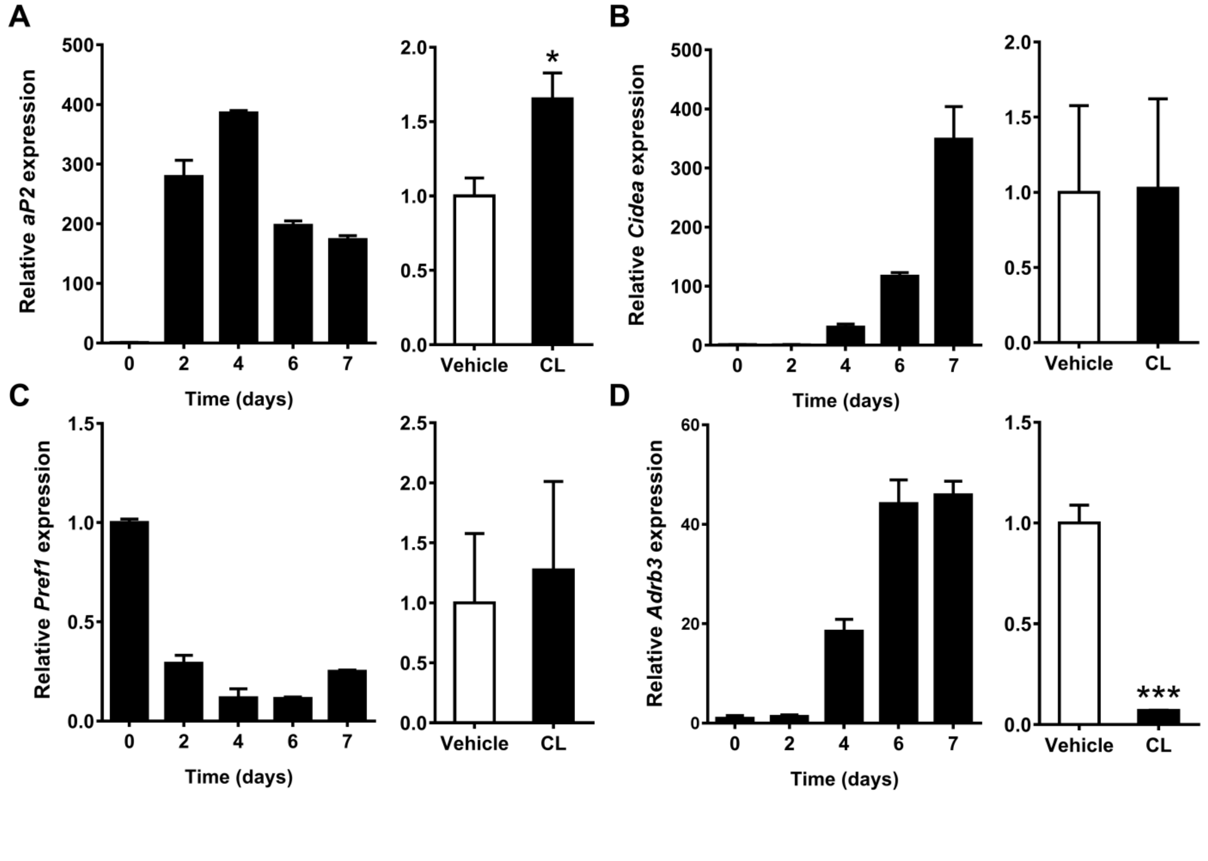

Appendix Figure S9. TUG-891 activates UCP1 by competitively interacting with GDP, but does not stimulate respiration through cAMP, ERK or AKT. Isolated mitochondria from WT and UCP1 KO mice (*n* = 4-5) were stimulated with TUG-891 or vehicle and mitochondrial oxygen consumption was measured (A, compilation of data shown in Figure 7B and C). TUG-891-induced mitochondrial respiration was measured in the presence of 1 or 3 µM GDP, in WT (B) and UCP1 KO (C) mitochondria (*n* = 3-5). Intracellular cAMP levels were measured in differentiated brown adipocytes pretreated with vehicle, TUG-891 (10 µM) or forskolin (10 µM) (*n* = 7) for 5 min. (D). Primary brown adipocytes (*n* = 2) were stimulated for 5 min with vehicle or TUG-891 (10 µM) and relative amounts of phosphorylated and total protein was determined by Western blotting. The amount of phosphorylated protein was normalized to the total amount of protein (E). Immortalized brown adipocytes (*n* = 5-6) were pretreated for 30 min with vehicle, U0126 (10 µM) or AKT1/2 kinase inhibitor (10 µM), after which the OCR was determined in a Seahorse XF24 analyzer. After three baseline measurements, either vehicle or TUG-891 (10 µM) was injected into the wells (F & G). Brown adipocytes were incubated with the calcium sensitive dye Fluo-4-AM for 1 h at RT, followed by live cell imaging with a spinning disk confocal (UltraVIEW Vox, PerkinElmer) and stimulation with TUG-891 (10 µM) with or without the presence of YM-254890 (0.1 µM) (H). F_1/F_0 represents peak fluorescence divided by baseline fluorescence. Data represent means ± SEM. * P < 0.05, * P < 0.01, *** P < 0.001 compared to the control group, according to the two-tailed unpaired Student's T test (A-B) or one-way ANOVA with Dunnett's post hoc test (C). The exact *P*-value for each significant difference can be found in Appendix Table S5.

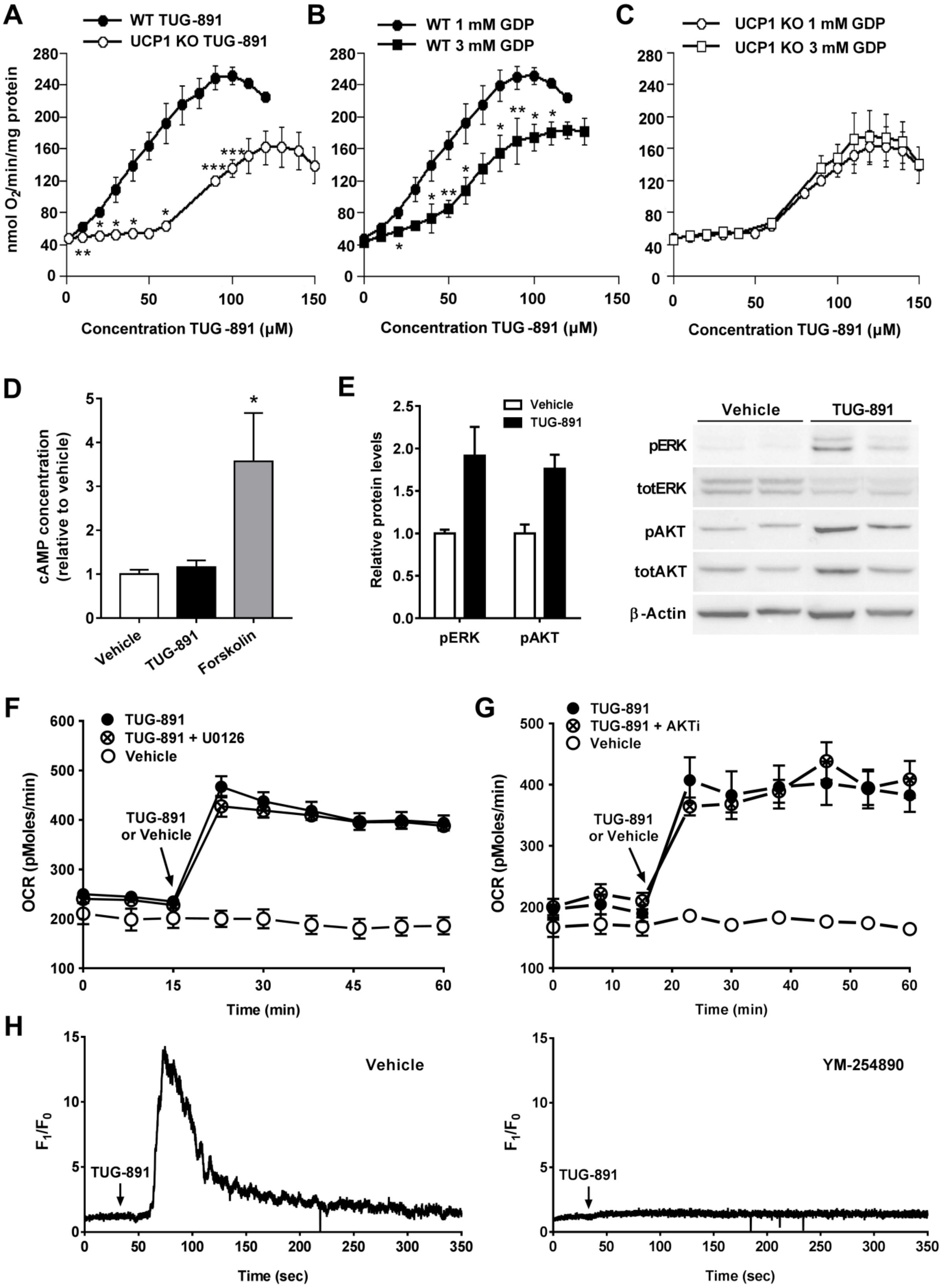
A
WT TUG-891
UCP1 KO TUG-891
nmol O2/min/mg protein
Concentration TUG-891 (µM)
B
WT 1 mM GDP
WT 3 mM GDP
Concentration TUG-891 (µM)
C
UCP1 KO 1 mM GDP
UCP1 KO 3 mM GDP
Concentration TUG-891 (µM)
D
cAMP concentration (relative to vehicle)
Vehicle
TUG-891
Forskolin
E
Vehicle
TUG-891
Relative protein levels
pERK
pAKT
Vehicle
TUG-891
pERK
totERK
pAKT
totAKT
β-Actin
F
TUG-891
TUG-891 + U0126
Vehicle
OCR (pMoles/min)
TUG-891 or Vehicle
Time (min)
G
TUG-891
TUG-891 + AKTi
Vehicle
OCR (pMoles/min)
TUG-891 or Vehicle
Time (min)
H
F1/F0
Vehicle
TUG-891
Time (sec)
YM-254890
TUG-891
Time (sec)
